# Plastic-Associated Chemicals: Late Lessons from Early Equilibrium Partitioning Science

**DOI:** 10.1021/acs.est.5c04383

**Published:** 2025-06-02

**Authors:** Heather A. Leslie, Annika Jahnke, Elisa Rojo-Nieto, Hans Peter H. Arp

**Affiliations:** † Heather Leslie Projects, Zamenhofstraat 108-408, 1022 AG Amsterdam, The Netherlands; ‡ Department of Exposure Science, Helmholtz Centre for Environmental Research GmbH - UFZ, Permoserstraße 15, DE-04318 Leipzig, Germany; § Institute for Environmental Research, RWTH Aachen University, Worringerweg 1, DE-52074 Aachen, Germany; ∥ Norwegian Geotechnical Institute (NGI), P.O. Box. 3930, Ullevål Stadion, N-0806 Oslo, Norway; ⊥ Norwegian University of Science and Technology (NTNU), N-7024 Trondheim, Norway

**Keywords:** plastic additives, microplastics, nanoplastics, plastic pollution, sorption, fugacity, chemical activity, weathering

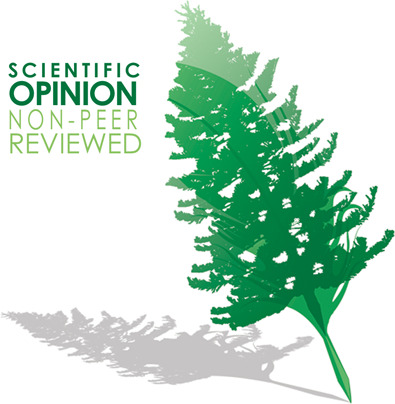

## Graphical abstract

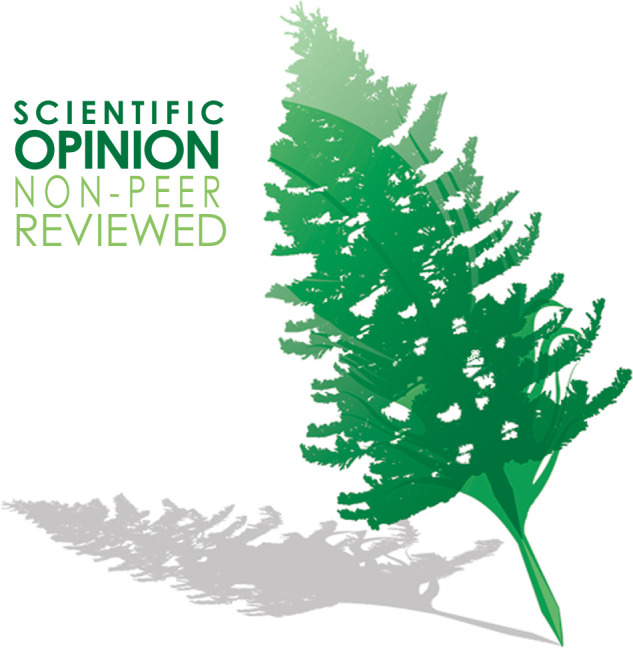

Concern about plastic pollution is driving research toward a better understanding of how plastic-associated chemicals may affect the environment and human health. A theoretical framework on how such chemicals leach, sorb, volatilize, and get transported would improve the understanding and advancement in this field, informing experimental design, predictions, and the interpretation of observations. But does such a framework already exist?

As an overarching theory, equilibrium partitioning (EqP) has been applied to study, model, and predict bioaccumulation, multimedia fate, and transport for decades. In our view, plastic-associated chemicals research could benefit from revisiting processes governing chemical partitioning using EqP theory. In the fields of passive sampling and passive dosing, EqP theory has already been extensively developed for polymer–chemical interactions.^1,2^

Early sentinels in the EqP field like René Schwarzenbach and the late Don Mackay taught generations of students to understand the physical organic chemistry of environmental pollutants, encouraged their readers to “find fugacity feasible, fruitful and fun”,^3,4^ and produced well-read handbooks.^5^ Their protégés continue the tradition of making physical chemistry concepts sound understandable and even intuitive. They have developed a mechanistic understanding of the behavior of organic chemicals interacting in multimedia systems, including plastics.

The complex behavior of chemicals associated with plastics can be understood as a surprisingly predictable diffusion and distribution behavior across the multimedia environmental stage. This body of EqP literature may be slightly off the beaten path, yet we consider it a genuine gold mine for plastic-associated chemicals research today.

## What Is EqP?

Chemicals are continuously diffusing through their surroundings and ultimately are distributed among the different phases (e.g., water, plastics, and biological membranes) in their environment, based on a process called partitioning. Given time, the chemicals tend to accumulate in and/or onto the phases present in such a way that they maintain a low-energy state in the system. EqP theory explains the relationship between the concentration of a chemical sorbed to plastics compared to its concentration(s) in the surrounding bulk phase(s), after reaching equilibrium.

A chemical’s partition coefficient, *K*, e.g., between plastic and water (=*C*_plastic,eq_/*C*_water,eq_), describes the ratio of the chemical’s concentrations in the two phases at equilibrium, at their lowest-energy state. *K* depends on the physicochemical properties of the chemical, the plastic, and the surrounding bulk phase(s). EqP enables us to predict where chemicals will flow and how concentrated they will be and can provide some clues about how fast the process will happen. This mechanistic understanding offers a way of reducing blind spots and head scratching.

To understand chemical partitioning to and from plastics, let us consider how chemicals can interact both inside a plastic item and on its surface. There can be “absorptive” interactions of chemicals inside the amorphous regions of the plastics and also “adsorptive” interactions on the plastic surface. For a chemical sorbing to a given plastic, *K*_plastic_ can be defined as the sum of absorptive interactions considering the “accessible, amorphous fraction of plastic” (*f*_amorphous_, unitless) and adsorptive interactions on specific surface area per mass (SSA, m^2^/kg, eq 1):

1 $$K_{\mathrm{plastic}}=K_{\mathrm{absorption}}f_{\mathrm{amorphous}}+K_{\mathrm{adsorption}}\mathrm{SSA}$$

Plastic materials (Figure 1a) range from being completely amorphous such that chemicals can freely diffuse in the entire polymer volume (*f*_accessible_ = 1; e.g., many silicone rubbers), over semicrystalline (*f*_accessible_ between 0 and 1; e.g., commodity plastics), to crystalline, i.e., no diffusion in the polymer (*f*_accessible_ = 0).^5^ For most small molecules, absorption is more relevant than adsorption; adsorption becomes more relevant for fully crystalline plastics, very thin films, fibers, and particles (<10 μm), for very short time scales before absorption can occur, or for macromolecules that are too large to diffuse into the amorphous regions of a plastic.

**Figure 1:**
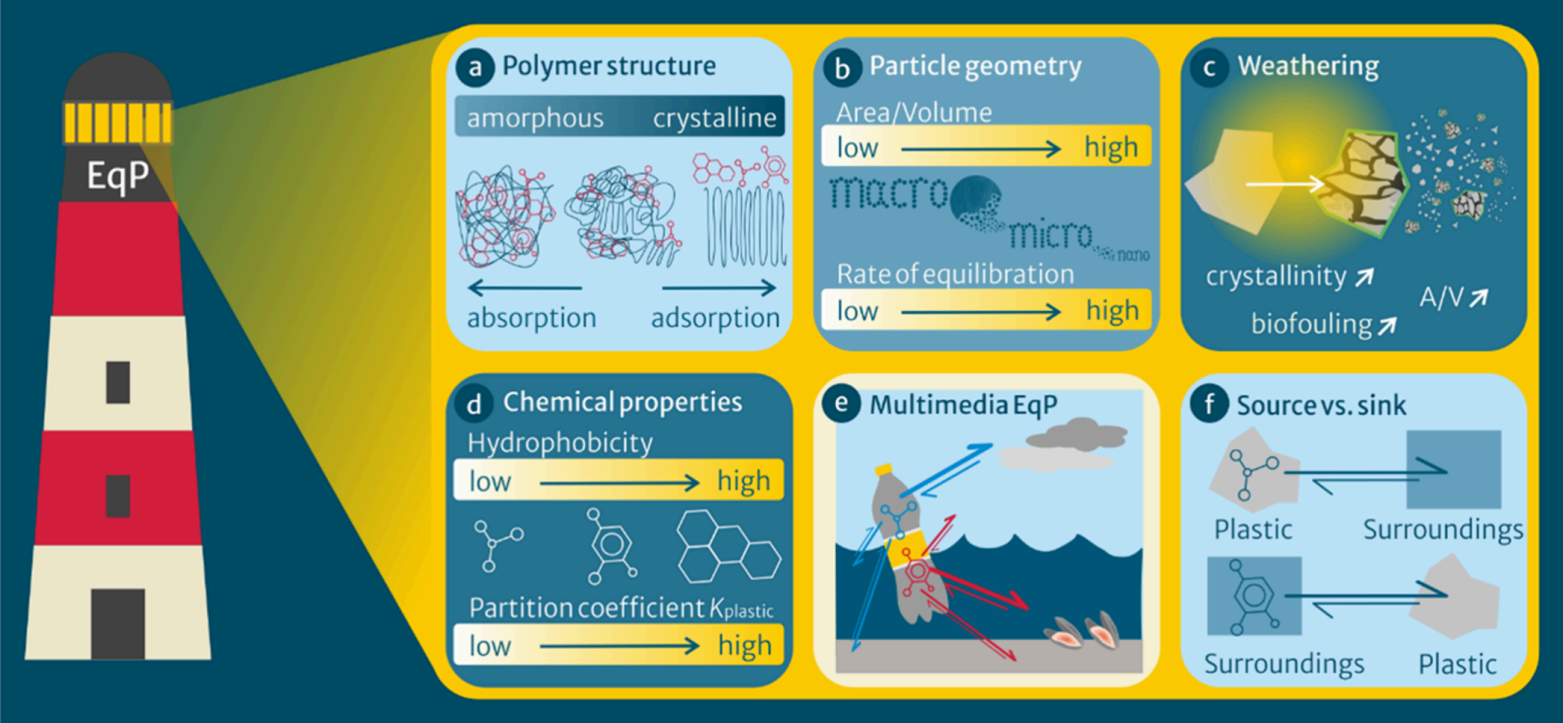
Equilibrium partitioning (EqP) sheds light on interactions between plastic-associated chemicals and plastics in various contexts: (a) different polymer structures and properties, (b) particle geometry, (c) weathering, (d) chemical properties such as hydrophobicity, (e) multimedia distribution, and (f) plastics as a source vs sink of chemicals.

Plastic particle size and shape affect kinetics (Figure 1b). First, adsorptive interactions are faster than absorptive processes, as only diffusion from the surrounding bulk phase to the plastic surface is needed. The larger the particle, generally the smaller the SSA, and the longer the diffusion pathways from the surface to the center of the plastic item to reach equilibrium for absorptive partitioning. Weathering processes (Figure 1c) alter crystallinity and increase brittleness; hence, they affect both absorptive and adsorptive processes. Greater crystallinity decreases *f*_accessible_ and diffusion rates, while surface cracking or biofilm growth affects *K*_adsorption_, SSA, and kinetics.

Plastic materials can leach plastic-associated chemicals with diverse polarities, sizes, and hydrophobicities to, e.g., water, whether that water is in aquatic environments, in the atmosphere, or inside organisms. Greater chemical hydrophobicity and molecular size act to increase *K*_plastic_ in water (Figure 1d). The aspects shown in Figure 1a–d will ultimately affect the partitioning behavior of plastic-associated chemicals in multimedia environments, where EqP can be reached between the plastic and air, water, sediment, or soil phases (Figure 1e). EqP predicts whether a plastic item will act as a “source” (i.e., leaching to surrounding media) or a “sink” (i.e., adsorbing or absorbing chemicals from the surroundings) for plastic-associated chemicals (Figure 1f).

Plastics can act as passive samplers when introduced into an environmental medium containing a chemical of interest, absorbing it until *K*_plastic_ is reached. Leaching will be observed again when the plastic is exposed to less contaminated media, until *K*_plastic_ in the new system is restored. This leaching is particularly important in terms of the release of hazardous chemicals, e.g., additives leaching or off-gassing from plastics, or plastic-mediated long-range transport from contaminated to pristine environments. The dynamic nature of surrounding environmental conditions as well as the weathering of plastics can influence EqP, but these challenges can be addressed.

## Limitations of EqP

EqP represents a steady state, which is achievable under laboratory conditions but rarely in the environment. Changing environmental conditions, be it fluctuations in concentrations, turbulence, light intensity, biofilm formation, surrounding media, or temperature, will affect chemical diffusion and degradation rates and drive plastic weathering processes that affect partitioning.^6^ EqP processes are slow for many chemicals associated with plastic. It can take months to years for very hydrophobic chemicals to partition to equilibrium concentrations if they have to diffuse through a water phase, and a stagnant boundary layer, to sorb to plastic, or to leach from it. In real-world situations, this equilibration can be further slowed by low temperatures or biofilm formation (Figure 1c).^7^ In contrast, adsorptive processes, such as the formation of an eco-corona of various metabolites on plastics when introduced to water and soil, can be quite fast.^8,9^ Bulk phase flows such as air movements, river water flows, etc., establish a dynamic system in which chemicals interact, for instance, chemicals in riverbed sediments partitioning between sediment and flowing surface water phases of constantly changing contaminant loads and turbulences.

Nevertheless, EqP remains a valuable tool as it defines the thermodynamic end point for plastic-associated chemicals. In the laboratory, we can measure this end point and the kinetic rates of reaching it. Furthermore, the way controlled changes in environmental conditions will alter the EqP end point and its kinetics can be tested. Through the framework of EqP as an end point, and clarifying sensitivity to environmental conditions, we gain a way of understanding and simplifying complex plastic–chemical interactions in changing environments. This understanding is achieved by isolating the steady-state condition, the kinetics of the processes, and factors that act to slow or prevent EqP from being reached.

## What Is in It for Plastics Researchers?

EqP gives us the theoretical framework and insights to help make sense of the complex experimental data coming out of plastics leaching studies carried out under various conditions, in terms of both the distribution of chemicals in the system and the kinetics of the chemical partitioning processes. These insights can have multiple implications, ranging from an improved understanding of the environmental fate of plastic-associated chemicals during long-range transport^10^ to better accounting for plastic sorption biases in bioassays^11^ to designing safer plastics and materials that leach less hazardous chemicals following Safe and Sustainable by Design (SSbD) principles.^12^ Understanding the distribution patterns and impacts of plastic-associated chemicals cannot be achieved without understanding EqP.
